# A Security Enhancement of the Precision Time Protocol Using a Trusted Supervisor Node

**DOI:** 10.3390/s22103671

**Published:** 2022-05-11

**Authors:** Waleed Alghamdi, Michael Schukat

**Affiliations:** School of Computer Science, National University of Ireland, H91 TK33 Galway, Ireland; michael.schukat@nuigalway.ie

**Keywords:** cyberattacks, IEEE 1588, PTP, security, time synchronization protocols

## Abstract

The Precision Time Protocol (PTP) as described in IEEE 1588–2019 provides a sophisticated mechanism to achieve microsecond or even sub-microsecond synchronization of computer clocks in a well-designed and managed network, therefore meeting the needs of even the most time-sensitive industrial and financial applications. However, PTP is prone to many security threats that impact on a correct clock synchronization, leading to potentially devastating consequences. Here, the most vicious attacks are internal attacks, where a threat actor has full access to the infrastructure including any cryptographic keys used. This paper builds on existing research on the impact of internal attack strategies on PTP networks. It shows limitations of existing security approaches to tackle internal attacks and proposes a new security approach using a trusted supervisor node (TSN), in line with prong D as specified in IEEE 1588–2019. A TSN collects and analyzes delay and offset outputs of monitored slaves, as well as timestamps embedded in PTP synchronization messages, allowing it to detect abnormal patterns that point to an attack. The paper distinguishes between two types of TSN with different capabilities and proposes two different detection algorithms. Experiments show the ability of the proposed method to detect all internal PTP attacks, while outlining its limitations.

## 1. Introduction

The precision time protocol (PTP) is widely used to provide time synchronization for many time-sensitive applications, for example, in telecommunications, industrial control, and financial services. PTP is deployed on local area networks, and it is the preferred protocol for sectors that need accuracies down to microsecond level or better [1]. However, PTP networks are vulnerable to cyberattacks that stealthily reduce the accuracy of time synchronization to undesirable levels for some clocks or an entire network.

Since PTP was introduced in 2002, the underlying IEEE 1588 standard has been revised twice to provide better accuracy and make the synchronization process more robust against attacks. The first improvement was released in 2008 (PTP version 2) [1], which introduced a new PTP-aware device called transparent clock that improves accuracy by better accounting for PTP packet transmission delays [1]. Although PTP version 2 (IEEE 1588–2008) introduced an experimental security extension (Annex K), it was shown that it is insufficient to deter PTP attacks, while it may be used to launch DoS attacks [2,3]. In addition, IEEE 1588–2008 is not backward compatible with the first version IEEE 1588–2002. In contrast, the latest version, IEEE 1588–2019, is backward compatible to IEEE 1588–2008. IEEE 1588–2019 can provide sub-nanosecond time transfer accuracy in a well-designed network [4]. Furthermore, it introduces multipronged security that combines a set of security mechanisms and configuration options listed in Annex P to provide PTP security. Annex P contains four prongs as follows [5]:

- Prong A (integrated security mechanism) describes the use of AUTHENTICATION TLV (type-length-value) that provide protection of PTP messages cryptographic integrity check values (ICV) based on symmetric keys.
- Prong B (PTP external transport security mechanisms) addresses the use of existing data link and network layer security protocols, i.e., IP Security (IPsec) and Media Access Control Security (MACsec), to protect PTP messages.
- Prong C (architecture mechanisms) highlights different redundancy techniques, i.e., redundant paths, redundant grandmaster, and redundant time systems (e.g., a PTP clock that has a GPS receiver as well as is synced to the grandmaster clock).
- Prong D (monitoring and management mechanisms) describes how a monitoring system can enhance PTP security.

However, research has shown that prong A and B cannot provide protection against some PTP attacks such as the delay attack [6,7,8]. Moreover, prong C does not provide comprehensive protection if an attacker simultaneously compromises all redundant systems [9], redundant paths [10,11], or redundant grandmasters [12].

Alghamdi et al. [13] investigated the impact of all PTP attack strategies as outlined in [7], considering the two most popular PTP daemons, PTP4l and PTPd, thereby highlighting the need for advanced attack detection methods.

This paper is an extension to this previous work and proposes a detection system that is aligned with prong D as described in IEEE 1588–2019 Annex P. The underlying idea is centered around analyzing data collected from all slave clocks in a network during the synchronization process and then monitor for anomalies caused by an attack. The proposed detection system can work efficiently on a device without an exact time reference (e.g., an unsynchronized stand-alone device), or on a dual clock device that has a PTP slave clock and an accurate unsynchronized local clock (e.g., a non PTP-synchronized device with an oven-controlled crystal oscillator), thereby providing different levels of capabilities in detecting attacks.

The main contributions of this paper are summarized as following: (1) proposing a central unit or trusted supervisor node (TSN) that collects the synchronization outputs from all monitored slave clocks and rearranges or groups the collected data to common synchronization cycles, (2) implementing two attack detection mechanisms depending on the attack scope (i.e., a subset of slave clocks or all slave clocks), (3) thereby specifying the attack strategy type, (4) and determining the possible attacker location within the network (i.e., the manipulated node that impacts all downstream nodes) using the lowest common ancestor method. In comparison to other monitoring systems, the TSN does not necessarily require another time reference to detect all PTP attacks, making it more attack resilient. In addition, security mechanisms are proposed to harden the TSN itself against malware infections and to secure communication between slave clocks and the TSN.

Section 2 presents a PTP overview, PTP security concerns, and existing PTP security measures. Section 3 highlights the TSN concept, including the data collection mechanism. An experimental testbed, which combines hardware and software required to perform the TSN role and to launch PTP attacks, as well as the conducted experiments, is presented in Section 4. A summary of results is discussed in Section 5, followed by a conclusion and future work in Section 6.

## 2. The Precision Time Protocol and State-of-the-Art Security Measures

### 2.1. PTP Overview

A PTP network may contain different types of clocks, i.e., ordinary clocks (OC), boundary clocks (BC), and transparent clocks (TC). An ordinary clock has only a single port that works either in a master state or slave state (slave clock). A single ordinary clock is in the master state and called the grandmaster clock (GM). It provides the time reference for the entire network. A boundary clock has multiple PTP ports, with one of them (facing the GM) in a slave state, while the others (i.e., downstream) are in the master state. A transparent clock is an intermediate node that compensates for PTP packets’ time crossing the device by measuring and adding the resident time to the *correctionField* value [14]. The GM is chosen among all ordinary clocks in a network using the best master clock algorithm (BMCA) that chooses the clock with the best attributes to become a master.

PTP can use two different mechanisms to measure the delay between PTP clocks, i.e., the End-to-End request-response mechanism (E2E) or the Peer-to-Peer mechanism (P2P). It also has two operation modes, i.e., one-step and two-step mode. The E2E delay mechanism uses the *Sync*, *Delay_Req*, *Delay_Resp*, and, if required, *Follow_Up* messages. In contrast, the P2P delay mechanism uses the *Pdelay_Req*, *Pdelay_Resp*, and, if required, *Pdelay_Resp_Follow_Up* messages [1].

**Figure 1:**
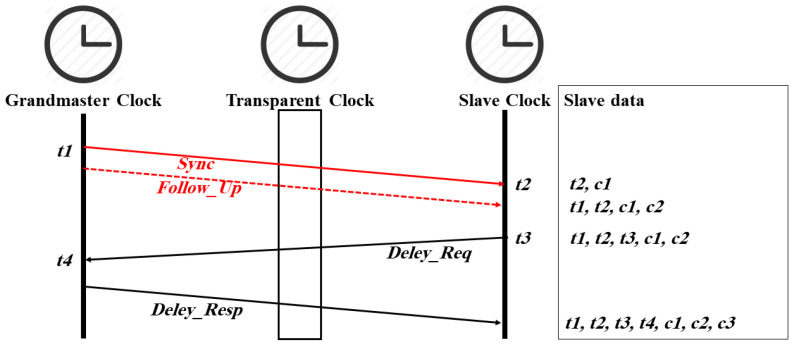
PTP timestamps and time synchronization messages in E2E delay mechanism.

**Figure 2:**
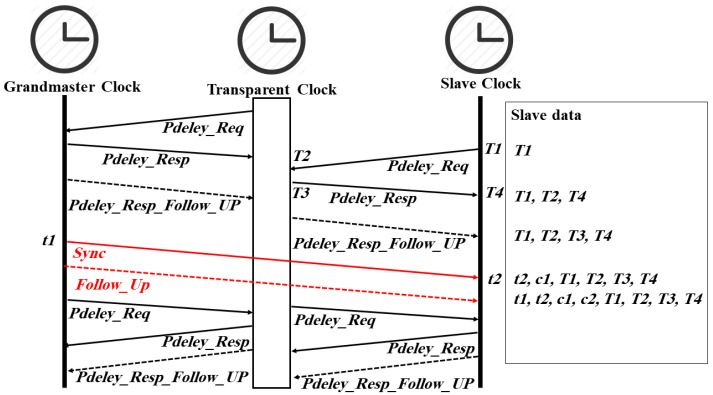
PTP timestamps and time synchronization messages in P2P delay mechanism.

Time synchronization in IEEE 1588 is based on the exchange of messages between the master and its slaves, as shown in Figure 1 and Figure 2. In the E2E mechanism (see Figure 1), the master sends a *Sync* message to slave clocks, which includes its departure time *t1*, while the slave clocks record its arrival time *t2*. If the master does not support hardware timestamping (one-step operation mode), the master sends a *Follow_Up* message that conveys *t1* to the slaves (two-step operation mode). Each slave sends a *Delay_Req* message to the master and records its departure time *t3*. The master records the arrival time *t4* and sends it back to the respective slave using the *Delay_Resp* message. If a transparent clock is located between the master and the slaves, the *correctionField* value of the *Sync* (*c1*), *Follow_Up* (*c2*), and *Delay_Req* (*c3*) messages will be updated when the messages are crossing the transparent clock device. Synchronization messages that belong to the same cycle share the same sync sequence id *syncID*, a 16-bit counter that is incremented with each cycle. Within a given cycle, the slaves have all timestamps required to calculate the offset and delay as follows [13]:

*offset = ((t2 − t1 − c1 − c2) − (t4 − t3 − c3))/2* (1)

*delay = ((t2 − t1 − c1 − c2) + (t4 − t3 − c3))/2* (2)

In the P2P mechanism (see Figure 2), the *Sync* and *Follow_Up* messages work in the same manner as the E2E mechanism, and the difference is only in the delay calculation. The path delay calculation is done by each P2P aware PTP port regardless of the port state (i.e., master or slave). This behavior helps correct the path delay immediately upon a network reconfiguration [1]. Figure 2 shows how PTP clocks exchange the required messages. The transparent clock firstly sends a *Pdelay_Req* message to the GM and records its departure time *T1*. The grandmaster clock receives and records the arrival time *T2* of this message. Next, the grandmaster sends a *Pdelay_Resp* message to the transparent clock that includes *T2* and records the departure time *T3*. After that, the grandmaster sends a *Pdelay_Resp_Follow_Up* message to the transparent clock that includes *T3*. Finally, the transparent clock generates T4 upon receiving the *Pdelay_Resp_Follow_Up* message. The path delay between the transparent clock and the grandmaster clock will be calculated at the transparent clock side as follows [1]:

*delay = ((T4 − T1) − (T3 − T2))/2* (3)

Depending on the applied operation mode (one-step or two-step operation mode), the calculated delay will be added to *c1* or *c2* before a *Sync* or *Follow_Up* message leaves the transparent clock. The slave clock will do the same to measure the link delay between the transparent clock and itself. The offset can then be calculated as follows [1]:

*offset = (t2 − t1 − c1 − c2) − slave link delay* (4)

### 2.2. PTP Attack and Attacker Types Overview

RFC7384 [8] discusses the security threats to PTP and distinguishes between two types, namely the internal and the external attack, which can be conducted either by a man-in-the-middle (MitM) or an injector attacker. Furthermore, Alghamdi [7] further distinguished between the simple and the advanced internal attack by determining the attack range, the attack impact on time synchronization, and the attack implementation. These attack/attacker types have different characteristics as follows.

#### 2.2.1. Simple Internal Attack

Here, the attacker has only access to a trusted component of the network, but no access to cryptographic keys. The attacker can launch several PTP attacks, such as the packet removal attack, packet delay manipulation attack, replay attack, or the DoS attacks [7].

#### 2.2.2. Advanced Internal Attack

The attacker has full access to a device, including the security keys used. This kind of attack can launch all PTP attacks as listed in [7].

#### 2.2.3. External Attack

The attacker resides outside the trusted network and has no knowledge of the security keys used. Such an attack is similar to a simple internal attack and takes place when PTP packets go through an untrusted network [7].

#### 2.2.4. Man-in-the-Middle (MitM) Attacker

The MitM attacker is located in a position that enables it to intercept a PTP packet and manipulate it in transit [7]. The packet content manipulation attack, the packet removal attack, the packet delay manipulation attack, the replay attack, and the DoS attack are examples of attacks that can be launched by the MitM attacker [7].

#### 2.2.5. Injector Attacker

The injector attacker is located in a position that enables it to generate network traffic [7]. The time source degradation attack, the spoofing attack, and the BMCA attack are examples of attacks that can be launched by the injector attacker [7].

### 2.3. Existing Security Mesures

#### 2.3.1. IEEE 1588 Annex K

Annex K was introduced with IEEE 1588–2008 as an experimental security extension that provides group source authentication, message integrity, and replay protection security using symmetric keys. It establishes a trust relationship using a challenge–response three-way handshake mechanism that relies on pre-defined keys shared by a group of devices or the entire domain [3]. Subsequently, various improvements have been proposed, including an improved handshake and replay counter [2]. The three-way handshake only increases traffic but provides no additional security [15]. In addition, an attacker can stage an Annex K-specific type of DoS attack or can get the symmetric keys from any of the existing clocks [16], allowing any PTP slave to masquerade as the grandmaster [3]. These and other flaws resulted in Annex K to be dropped in favor of other cryptographic protocol security extensions as outlined below [7].

#### 2.3.2. E2E Protocol Security (IPsec)

In IP networks, IPsec provides End-to-End (E2E) protection by encrypting and authenticating IP packet payloads, and by authenticating the non-alterable fields of the IP header. It supports a transport mode between endpoints and tunnel mode between two security gateways. IPsec can be used to prevent some external attacks, such as the packet content modification attack and the replay attack. Although this approach is robust as no rogue intermediate node can maliciously manipulate the payload of a packet, it may reduce PTP’s synchronization performance as it prevents intermediate TCs from updating the *correctionField* value. In addition, in authentication-only mode, IPsec does not fully prevent MitM or DoS attacks, since the PTP payload is not encrypted and can be easily manipulated. In contrast, encrypting PTP packets causes extra latency and jitter that negatively influences the synchronization performance [7].

#### 2.3.3. P2P Protocol Security (MACsec)

MACsec is a layer-two security protocol based on IEEE 802.1X, which specifies a session initiation and key management, and IEEE 802.1AE, which specifies the encryption and authentication protocol. The security architecture of MACsec uses a hop-by-hop authentication and encryption approach, where packets are decrypted and validated at each network node and then encrypted and authenticated again before being moved forward, therefore allowing TCs to update the *correctionField* value. MACsec can protect the connection of trusted nodes, but cannot deter attacks that are initiated by the same trusted nodes. This gives an opportunity to the advanced internal attacker, who resides in such a trusted node, to launch a PTP attack (e.g., a packet content modification attack) and therefore degrading the synchronization accuracy [7].

#### 2.3.4. Authentication Type-Length-Value (TLV)

IEEE 1588–2019 uses an AUTHENTICATION TLV to share security-related information required to calculate the integrity check value (ICV) that is used for integrity and authenticity verification purposes [17]. This TLV is attached to all PTP messages to be secured. A secret key is used to create a unique ICV that is appended to a PTP message [17]. IEEE 11588–2019 provides two types of AUTHENTICATION TLV, namely immediate and delayed security processing. The former uses a key management protocol, e.g., Group Domain of Interpretation (GDOI), to share all security parameters required to calculate the ICV and therefore process the AUTHENTICATION TLV before the PTP message content is further processed. For example, master and slave clocks can use immediate security processing to protect the outgoing messages by creating and appending AUTHENTICATION TLV and ICV to these messages and verify them at each PTP clock upon reception [17]. In contrast, the delayed security processing depends on an associated key management protocol, e.g., Time Efficient Stream Loss-Tolerant Authentication (TESLA), which supports delaying the distribution of required security parameters, including the secret key. Here, the master uses a specific secret key to protect the outgoing messages and the slave receives and buffers these messages for later verification. Eventually, the master uses a new secret key and discloses the old one, allowing the slaves to verify the integrity of the buffered messages [17]. Both immediate and delayed security processing can be included in one PTP message by attaching two AUTHENTICATION TLVs. The first AUTHENTICATION TLV is used to authenticate the PTP message sent by the originator, while the second AUTHENTICATION TLV is used to protect the mutable fields, e.g., *correctionField*. In this case, the mutable parts of a PTP message are authenticated by the immediate security processing (i.e., a TC can update the *correctionField* value) while the other parts are authenticated by the delayed security processing [4]. However, the AUTHENTICATION TLV has the same limitation as IPsec and MACsec, i.e., it is vulnerable to delay attacks and the advanced internal attacker [6].

#### 2.3.5. Multiple Paths

The dynamic nature of network behavior forms a challenge to time synchronization. The accuracy of clock synchronization depends on the steadiness and symmetry of propagation delays in the uplink and downlink direction between the master clock and its slave clocks. However, computer networks can show path asymmetries or variable network latencies. This can be addressed by using multiple alternative paths on local area networks (LAN) by using VLAN [10] or via the Parallel Redundancy Protocol (PRP) and High-availability Seamless Redundancy (HSR) [11]. Path redundancy does also improve robustness against MitM attacks [7], while Han [18] and Neyer [19] showed that multiple packet propagation paths can detect delay attacks by comparing the offset values computed by each slave port. However, an attacker can manipulate all paths and therefore desynchronize the slave clocks [7].

#### 2.3.6. Redundant Grandmaster

Redundant grandmasters can help detecting the byzantine failure, in which the grandmaster sends an inaccurate time reference because of an attack or a malfunction. The redundant grandmaster(s) compare GM timestamps with their own time reference and determine a new GM if a threshold is exceeded. Again, an attacker can target all redundant grandmasters and, therefore, the attack remains unnoticed [7].

#### 2.3.7. Protocol Redundancy

Another possible way to encounter byzantine failures is to combine NTP and PTP time synchronization services on slaves by computing NTP time synchronization messages from several NTP servers as well as PTP synchronization messages send by the GM. If the calculated PTP and NTP offsets exceed a configurable threshold, it is assumed that the GM is erroneous, and a slave clock will be synchronized using NTP. However, the NTP and PTP protocols are vulnerable to the same attack strategies, such as packet content modification attack, replay attack, and DoS attack [7].

#### 2.3.8. De-Militarized Zone

The De-Militarized Zone (DMZ) [20] is a security method that is acting as the first line of defense to protect internal network infrastructure by containing and exposing only an organization’s external-facing services to the Internet. It is useful for a network that needs to share devices or endpoints publicly (e.g., web servers). As such, it does not provide protection against an internal attacker who resides in a trusted network [7].

#### 2.3.9. Monitoring Systems

Since the attack impact can be noticeable by slave clocks regardless of preventive methods used to protect a PTP network, collecting and analyzing data from these slaves forms the cornerstone to detect such attacks [7]. Annex P prong D of IEEE 1588–2019 outlines such monitoring and management mechanisms. The underlying idea is that monitoring the performance of the PTP network can provide a good indication of potential PTP attacks, particularly the delay attack. For example, collecting the link delay between nodes (P2P mechanism) or between master and its slaves (E2E mechanism) using a central management system can help detecting a potential attack. Furthermore, a central management system can detect unexpected offset jumps or large offset corrections that reflect a large drift of a local clock oscillator. IEEE 1588–2019 (Annex J) provides a list of potential parameters and statistics counters that can be used to monitor the performance of a PTP network and PTP clock operation. These parameters include the master–slave delay, the slave–master delay, the mean path delay, and the offset from the master. Annex J suggests calculating the average, maximum, minimum, and standard deviation of each of these parameters over 15-min and 24-h intervals. Moreover, Annex J counts the announce messages sent/received by/from the GM, the announce messages received from foreign masters, *Sync* messages sent/received by/from the master, *Follow_Up* messages sent/received by/from the master, *Delay_Req* that have been sent/received, and *Delay_Resp* that have been sent/received in 15-min and 24-h intervals [4]. However, Annex J does not outline how to compare these statistics, nor does it determine a security threshold that can be used to trigger an alarm.

### 2.4. Related Work

In line with prong D, some researchers have proposed PTP attack detection methods using different ways such as comparing the slave offset in a reference clock, round trip delay (RTD) measurements, and sudden changes in the offset or the delay values obtained by the network clocks. Moussa et al. [21,22] proposed a detection method based on another time reference that is capable of collecting timestamps from slaves and comparing them with a reference timestamp in order to detect PTP attacks. Nevertheless, their proposal does not provide protection against all potential PTP attacks. In addition, their solution is vulnerable to attacks by an APT (Advanced Persistent Threats) [23,24], where the threat agent simultaneously manipulates all time references in a network or manipulates all timestamps sent by slaves. Using the same approach (i.e., comparing the slave offsets against a reference clock), Moradi et al. [25] suggested dividing all PTP clocks in a network into different blocks. Each block contains at least one master clock (i.e., GM or BC) and calculates the offset between this master and the slaves, before sending it to an analysis unit. If the offset is a non-zero value, the analysis unit will announce an attack on that block. However, achieving a zero offset in a PTP network is very difficult to achieve even in the most optimal PTP network. In addition, the proposal focuses on the delay attack only. Ullmann [24] suggested another detection method that calculates the uplink/downlink delays of each path and stores these values as a reference in the master. A delay attack is detected when a measured uplink/downlink path delay has a significant difference from the reference value. However, normal network traffic can encounter some delay variations, which may result in a false alarm. In addition, the proposal does not provide protection against the other attack types. Similar work has been proposed by [26] (i.e., using RTD measurement). Lisova et al. [26] suggested monitoring messages sent by the grandmaster at the slave side. The authors assumed that these messages are received in equal intervals unless an attack is present. However, any computer network is prone to path asymmetry and variable network latency, depending on the nature of the underlying network [27,28] and, therefore, false alarms can be raised. In addition, there is no guarantee that the grandmaster will always send messages in regular intervals, instead differences in the order of milliseconds are possible. With regards to the last detection approach, i.e., sudden changes in the calculated offset/delay values, the underlying idea is that an attack will be announced when a sudden change of the delay/offset values is observed by at least one slave clock [25]. However, it has been shown that a time source attack does not result in incremental delay calculations and therefore make such kind of attack detection insufficient [13].

This paper proposes a trusted supervisor node (TSN) that acts as a central management system. It provides two different algorithms consistent with prong D that are able to detect all PTP attacks as listed in [7]. In contrast to previous approaches, this proposal does not necessarily require the existence of another time reference to detect all PTP attacks. In addition, it takes advantage of more PTP clock parameters to identify and classify attacks. More details and comparisons between TSN and the other approaches, including Prong D, are further discussed below.

## 3. The Trusted Supervisor Node Concept

In line with the ideas of Annex P Prong D, a detection system is proposed to detect PTP attacks using a monitor unit. The underlying idea of the detection system is that although individual slave clocks are intrinsically inaccurate and likely to drift [29], a subset of slaves may show a statistically significant deviation in their synchronization outputs if they are simultaneously exposed to manipulation [2]. The trusted supervisor node (TSN) is proposed to act as a monitoring unit for all slaves in a PTP network.

The TSN is an isolated and protected device that can be either:

- Type-A: a stand-alone device with a normal unsynchronized clock oscillator.
- Type-B: a dual clock device. Here, the TSN contains an unsynchronized accurate clock with very predictable and stable drifts, e.g., an oven-controlled crystal oscillator, and a normal PTP synchronized slave clock.

Each TSN type has different capabilities in detecting PTP attacks: since a Type-A TSN does only have access to the collected data from the monitored slave clocks, it can only use statistical variations in these data to detect a PTP attack. A Type-B TSN exposes itself as potential attack target. Having two independent clocks in a TSN device (i.e., a synchronized slave clock and an unsynchronized local clock) provides more baseline data to detect the most silent attack, i.e., the time source degradation attack.

The TSN, as a protected endpoint, may use TPM (Trusted Platform Module) to store encryption keys/hash values that are utilized for hardware validation and to authenticate its firmware when booted, thereby hardening the system against malicious malware injections or hardware manipulations that would render its operation useless. Data communication from slaves is authenticated via digital certificates using a public key infrastructure, thereby avoiding the problem of MitM and injector attacks aimed to falsify the slaves’ clock information by applying best industry practices. These certificates are exchanged using a handshake protocol and mutually authenticate the other side’s cert to build a trust relationship. In this process, the TSN and slaves agree on pairwise shared private keys, which are subsequently used to encrypt/authenticate all data coming from a slave. While this process requires additional computational resources, e.g., the integration of a TLS/HTTPS protocol stack, it does not limit the detection capabilities of the TSN. The received and authenticated slave clock data, transported in slave clock status messages (SCSMs), are processed by the TSN and stored locally. Using this approach, the injection, manipulation, out of order delivery, and replay of (unsolicited or stale) SCSMs are easily detected. Packet transmission latencies or jitter (deliberately caused or a result of network traffic) do not matter either. Furthermore, each slave can use a combined digital/attribute certificate [30] to report its clock characteristics including clock stability and typical clock drifts to the TSN, which can be subsequently used to complement a slave clock’s baseline parameters outlined further below.

The TSN collects data from authenticated slave clocks and analyses them in real-time using time series analysis methods, allowing it to detect an attack in real-time. The baseline parameters of every slave are locally calculated, transmitted, and processed using the *slave monitor client* program and the *data concentrator server* program. The slave monitor program is installed on each slave clock endpoint, and its role is, after a successful mutual authentication, to send per synchronization cycle a SCSM to the TSN, which includes:

- A unique slave identifier *slaveID* (i.e., the authenticated slave’s IP address);
- The latest offset and delay values calculated by the slave daemon (taken from the PTP log file);
- The difference between the arrival time of the last received *Sync* message (*t2*) and the momentary delay value as calculated by the PTP slave, i.e., the estimated time of *Sync* packet departure from a slave’s perspective (estimated master timestamp—*EMT*);
- Key data from the latest *Sync*/*Follow_Up* message sent by the GM, i.e., the *SyncID*, GM timestamp (*t1*), *correctionField* content (*c1* or *c2*), the grandmaster clock ID (*grandID*), and the sync interval value (*syncInterval*) in second scale.

In contrast, the data concentrator program is installed on the TSN device, where it collects, authenticates, and analyzes SCSM from all slaves in real-time after the aforementioned initial mutual authentication with each monitored slave.

The TSN requires some baseline data before going into the monitoring stage, as every device/network link may have unique characteristics. In the conducted experiments, these data are collected during an initial calibration period prior to an attack. All slave clocks have the same *syncInterval*, and *syncID* wrap-arounds are not dealt with. It is assumed that there may be one or more attackers performing possibly multiple attacks simultaneously.

Since PTP attacks may target synchronization packets in transit or the grandmaster clock itself, the TSN provides two different attack detection algorithms that work with both TSN types with minor restrictions as further outlined below. The baseline determination stage and the attack detection algorithms are as follows.

### 3.1. Baseline Determination Stage

During this initialization stage, for each monitored slave that presents itself to the TSN (i.e., initiates an authentication handshake), a trust relationship is established. Subsequently the TSN determines the slave’s *slaveID*, *grandID*, and the *syncInterval* value, and processes over a configurable period (C_P_) incoming SCSM, which are stored in a circular buffer of size N_B_.

Once the circular buffer contains N_B_ elements, the TSN calculates/updates with each new SCSM arriving:

- The largest observed *correctionField* value *c1* or *c2* (C_max_);
- The moving average of delay, as calculated by the slave’s PTP daemon, thereby updating an upper and lower delay average value (Delay_max_ and Delay_min_);
- The maximum and minimum observed offset values calculated by the PTP daemon (Offset_max_ and Offset_min_). Here, the TSN determines and eliminates offset outliers using a significance test based on a configurable z-score [31] first, before updating both values. This pre-selection is necessary, as PTP daemons do occasionally report intermittent offset spikes, for example, because of momentary network congestions or an instability of a slave clock frequency.

Finally, two threshold values are to be set that are required for both detection algorithms:

- The acceptable timestamp difference (T_Δ_). For a type-A TSN, this is the maximum tolerated difference between any two *EMTs* calculated by slaves for the same synchronization cycle (*SyncID* is used as an identifier); additionally, for a Type-B TSN, T_Δ_ is the maximum tolerated difference between its slave clock and the TSN clock.
- The minimum number of consecutive suspicious SCSM (N_SCSM_) before an attack is considered.

**Figure 3:**
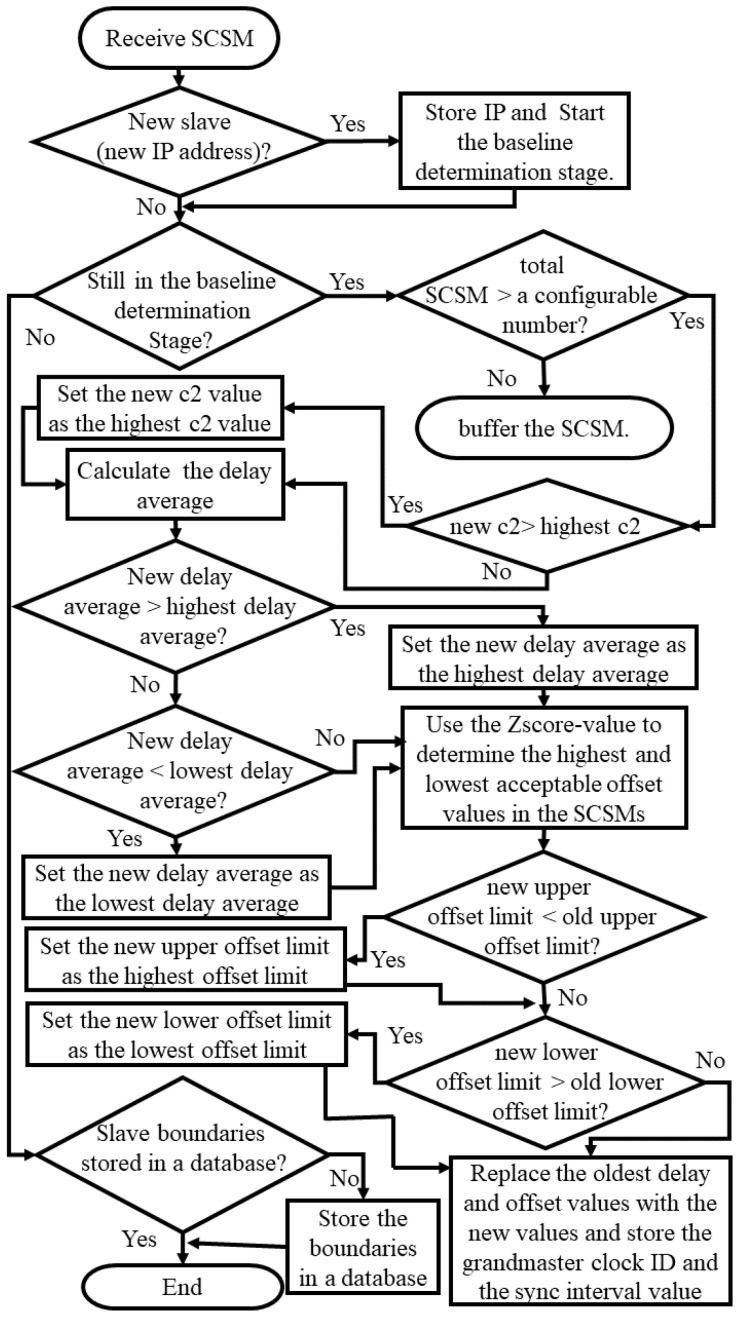
The baseline determination stage flowchart.

Figure 3 shows a summary of the baseline determination stage process and how both TSN types determine the slaves’ baselines parameters.

After completing the baseline determination stage for a given slave, all parameters are stored in a hash table with *slaveID* as key, and the TSN enters the slave monitoring stage.

### 3.2. Slave Monitoring Stage

Since all slaves use the same *syncInterval*, SCSMs are expected to arrive at the same average rate, but inter-arrival times may differ because of high slave CPU loads or network congestion. In addition, SCSM may be duplicated (i.e., SCSMs that have the same *syncID* and *slaveID*) because of a PTP replay/spoofing attack. Therefore, the TSN needs to handle both as further described below.

Incoming SCSMs are buffered and subsequently processed based on their *syncID*, with X denoting the current synchronization cycle to be considered. Packets that cannot be authenticated or are stale indicate a SCSM modification/fabrication, a replay, or a DoS attack on the TSN itself, and an alarm will be raised.

At the beginning of the monitoring stage, the TSN collects SCSM for three sync intervals starting from the arrival of the first SCSM. At this point (and subsequently in one-synchronization cycle intervals), the TSN moves all authenticated SCSMs from the buffer into different buckets based on their *syncID*. The buckets are as follows:

- staleBucket: contains SCSMs that have stale *syncID*s (*syncID* < X). A non-empty bucket indicates a very crude replay/spoofing attack on one or more slaves. After determining the potential attacker location using the LCA method (see below), an alarm is raised.
- activeBucket: contains all SCSMs to be processed next (*SyncID* == X).
- nextBucket: contains SCSMs with *syncID* > X. After the TSN processes the content of activeBucket, its content will be replaced with SCSMs of the next cycle (*syncID* = X + 1). If there are no such SCSM in the buffer (the result of a DoS attack or a network-wide synchronization fault), an alarm will be raised. Remaining SCSMs with a larger *syncID* than expected (*syncID* > X + 3) indicate a very crude replay/spoofing attack, resulting in the same action as with staleBucket.

Subsequently, the TSN processes the content of activeBucket using two separate algorithms in parallel as follows.

#### 3.2.1. Synchronization Attack Detection

The TSN processes the activeBucket content by updating each slaves’ delay moving average and extracting *EMT*, *c1* (or *c2*), and *t1*. While a temporary congestion or instability of the network may affect a slave’s clock synchronization momentarily, a deliberate attack must be executed continuously and affecting specific synchronization messages [7,13]. Therefore, a slave is deemed to be attacked, if N_SCSM_ consecutive SCSM show an increased *c1* (or *c2*) value (>C_max_), and/or if the calculated delay average is outside the established Delay_max_ and Delay_min_ boundaries. Compared to [24], this approach decreases the probability of false positive alarms, as once-off glitches are ignored. In addition, the TSN uses *EMT* and *t1* to further detect PTP attacks as further described below, which distinguishes between different attacks as follows.

1. Replay and spoofing attack

Replay and spoofing attacks have the same impact on slave clocks, therefore they are merged into one section. Beside the aforementioned *syncID* validation, the TSN raises an alarm if the activeBucket has more than one SCSM with the same *slaveID* (lines 28–30, and 76–79 in Algorithm 1).
**Algorithm 1:** Synchronization attack detection1**Input**: activeBucket2**Output**: messages indicating attack types, their root locations, and manipulated slaves if any3*delayMax*:Hash table containing the upper delay averages of all slaves (Delay_max_)4*delayMin*:Hash table containing the lower delay average of each slave (Delay_min_)5*delaySample*:Hash table containing lists of the last N_B_ delay values of each slave6*delay*:Hash table containing each slave’s *delay* value extracted from its SCSM 7*cMax:*Hash table containing the largest correction field value of each slave as previously determined (C_max_)8*c*:Hash table containing the *c1*/*c2* values extracted from its SCSM9*t1*Hash table containing current *t1* values extracted from SCSMs10*EMT*:Hash table containing current *EMT* values extracted from SCSMs11*IP*:A list containing the *slaveID* of all registered slaves 12*IP2:*A list containing the *slaveID* extracted from SCSM13*NSCSM:*Threshold value (N_SCSM_) as set before14*delayAverage:*Hash table containing the new delay average of each slave 15*TΔ:*The acceptable maximum difference between *EMTs* as determined before16*DoS*Hash table containing different counters based on different *slaveID*s attacked by DoS attacker (initialized with 0s)17*content1*Hash table containing different counters based on different *slaveID* attacked by packet content attacker (*t1*)18*content2*Hash table containing different counters based on different *slaveID* attacked by packet content attacker (c) (initialized with 0s)19*replaySpoofing*Hash table containing different counters based on different *slaveID* attacked by replay attacker or spoofing attacker 20*Delay*Hash table containing different counters based on different *slaveID* attacked by delay attacker (initialized with 0s)21*unknown*Hash table containing different counters based on different *slaveID* attacked by unclassified attack 22*LCA:*A method to determine and print the lowest common ancestor for each attack type23*baseline1**t1* value of the first slave that is not in *replaySpoofing*24*baseline2**EMT* value of the first slave that is not in *replaySpoofing*25*Set all values in content1, replaySpoofing,* and *unknown* to 0s26**for** *i =* 0 **until**
*IP.size −* 1 **do**27**     for** *j = i +* 1 **until**
*IP.size −* 1 **do**28**        if***IP[i]* = = *IP[j]*
**then**29**              ***replaySpoofing[IP[i]] = NSCSM +* 130**        end if**31**      end for**32**        if***replaySpoofing[IP[i]] ==* 0 **then**33            Replace the oldest *delay* in *delaySample[IP[i]]* with the *delay[IP[i]]*34**        end if**35**end for**36**for** *i =* 0 **until**
*IP.size −* 1 **do**37**     for** *j =* 0 **until**
*IP2.size −* 1 **do**38**        if***IP[i]* = = *IP2[j]*
**then**39            *DoS[IP[i]]* = 041                 *delayAverage[IP[i]]=*(sum of *delaySample[IP[i]]*/N_B_)42            break43         **else if**
*j* = = *IP2.siz*e − 1 **then**44                 *DoS[IP[i]]++*45**         end if**46**      end for**47**end for**48**for** *i =* 0 **until**
*IP2.size −* 1 **do**49**      if***replaySpoofing[IP2[i]] =* 0 **then**50*          baseline1 = t1[IP2[i]]*51*          baseline2 = EMT[IP2[i]]*52          break53**      end if**54**end for**55**for** *i =* 0 **until**
*IP2.size −* 1 **do**56**   if** *replaySpoofing[IP2[i]] ==* 0*  *
**then**57     **if** *baseline1* < > *t1[IP2[i]]*    **then**58*             content1[IP[i]]* = *NSCSM* + 159**     end if**60     **if** *c[IP2[i]]* > *cMax[IP2[i]]* **then**61*             content2[IP2[i]]++*62**     else**63*             content2[IP2[i]] =* 064**     end if**65      **if** *delayAverage[IP2[i]]* > *delayMax[IP2[i]]* **or**
*delayAverage[IP2[i]]* < *delayMin[IP2[i]]* **then**66*             Delay[IP2[i]]++*67**      else**68              *Delay[IP2[i]] =* 069**      end if**70    **if** abs (*baseline2* − *EMT[IP2[i]]*) > *T*Δ **then**71*             unknown[IP2[i]]* = *NSCSM* + 172**    end if**73**  end if**74**end for**75**for** *j =* 0 **until**
*IP.size −* 1 **do**76     **if**
*replaySpoofing[IP[j]]* > *NSCSM* **then**77**           print** “replay/spoofing attack”78**           print***replaySpoofing*79*           LCA (replaySpoofing)*80      **else if**
*DoS [IP[j]]* > *NSCSM* **then**81**           print** “DoS attack”82**           print***DoS*83*           LCA (DoS)*84      **else if**
*content1[IP[j]]* > *NSCSM* **then**85**           print** “content attack (*t1*)”86**           print***content1*87*           LCA (content1)*88      **else if**
*content2[IP[j]]* > *NSCSM* **then**90**           print** “content attack (c)”91**           print***content2*92*           LCA (content2)*93      **else if**
*Delay[IP[j]]* > *NSCSM* **then**94**           print** “delay attack”95**           print***delay*96*           LCA (delay)*97      **else if**
*unknown[IP[j]]* > *NSCSM* **then**98**           print** “unknown attack”99**           print***unknown*100*           LCA (unknown)*101**      end if**102**end for**

2. Denial of service (DoS) attack

A DoS attacker, located between the GM and the targeted slaves, can interfere with clock synchronization in different ways. Firstly, changing the *domainNumber*, and *versionPTP* fields in *Sync* messages causes a slave’s clock to go into free-running mode [7]. In addition, an attacker can intercept all *Sync*/*Follow_Up* messages and prevent them from reaching their destination. Since SCSM messages are linked to completed synchronization cycles, the TSN keeps track on missing SCSM per slave and raises an alarm if the threshold value N_SCSM_ is exceeded (lines 43–45 and 80–83 in Algorithm 1). This threshold is required to limit false positive alarms, as PTP uses the UDP transport layer protocol, which does not compensate for occasional packet losses.

3. Packet content manipulation attack

Here, the TSN checks and compares synchronization information of all slaves for inconsistencies that point to a content manipulation that took place along the path between the GM and the slaves. This includes differences in *t1* values (which must be identical), and excessive *c1* or *c2* values that are larger than C_max_ (see lines 57–64 and 84–92 in Algorithm 1). Since intermittent network congestion can make PTP messages reside in a TC longer than usual, the N_SCSM_ threshold reduces the probability of false positive alarms. Changes of the *versionPTP* or *domainNumber*, which cause a slave clock to go into free-running mode, are already dealt with in the DoS attack section.

4. Packet delay manipulation attack

Local clock offsets correlate to asymmetric uplink/downlink delays, which result in increased delay calculations of an affected slave. Subsequently, the TSN checks for each slave if the measured delay exceeds the previously determined Delay_max/_Delay_min_ boundaries for more than N_SCSM_ SCSMs (see line 65–69 and 93–96 in Algorithm 1), thereby accommodating again intermittent network uplink/downlink delay asymmetries because of traffic volumes.

Both TSN types also provide an additional verification to detect any unknown attack which targets a subset of slave clocks, by calculating the difference between the slaves’ *EMTs*. If their difference exceeds T_Δ_, an alarm will be raised (see line 70–72 and 97–101 in Algorithm 1). In addition, a Type-B TSN compares its synchronized PTP clock with its local clock and immediately raises an alarm if the difference between its clocks exceeds T_Δ_. Detecting an attack using T_Δ_ means that an attacker has manipulated some slaves using a new attack strategy or has kept an attack always below the N_SCSM_ threshold. This threshold, while making the algorithm more robust to compensate for glitches, also allows an attacker to interfere with the synchronization for just less than N_SCSM_ cycles, for example, by manipulating the *c1*/*c2* value. Once the attack is temporarily paused and normal *c1*/*c2* values below C_max_ are reported again, the TSN resets its counters. Such an attack will eventually desynchronize a slave clock and affect its *EMT*. Hence, the *EMT* comparison will detect all attacks that cannot be recognized by other comparisons.

The proposed algorithm is able to detect and track simultaneous attacks on multiple groups of slaves. As a set of attacks may simultaneously target one or more slaves, both TSN types are able to distinguish between them by dedicating a counter for each attack including the unknown attack and announce the attack when its counter exceeds the N_SCSM_ threshold.

**Figure 4:**
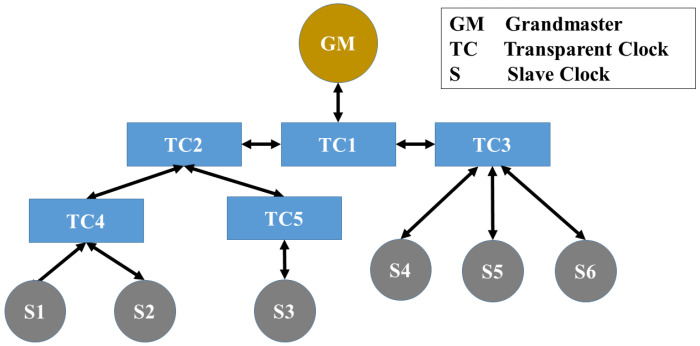
A simple PTP network.

Since slave clocks can be interpreted as leaves in a tree with the GM as the root, and network routers, switches, BC and TC as inner nodes, the lowest common ancestor algorithm (LCA) [32] can be used to identify the closest common ancestor node where the attack most likely takes place. Figure 4 shows a PTP network that contains one grandmaster, 5 transparent clocks, and 6 slave clocks. If a MitM attacker executes a delay attack only on S1 and S2, which is subsequently detected by the TSN, the LCA algorithm will return TC4 as the lowest common ancestor as the probable location of the attacker. However, calculating the LCA during a packet content manipulation attack or unknown attack will return the lowest common ancestor either of the attacked nodes or the unaffected nodes. This is because the TSN picks one (manipulated or not manipulated) SCSM as the baseline for its analysis. In addition, if two identical attacks are launched from inner node siblings, the algorithm will incorrectly return their parent as the location of the attacker.

#### 3.2.2. Time Source Attack Detection

The BMCA attack or the time source degradation attack targets the time synchronization source, and as such affects all slave clocks. It has been already established that both attacks cause an instantaneous spike of the offset error of manipulated slaves [7,13]. Hence, the emergence of such behavior in all slave clocks is a significant indicator for such an attack and is described in the following:

1. Time source degradation attack

A time source degradation attack may cause an instantaneous and simultaneous spike of the offset error calculated by each slave clock, which correlates to the size, the increment, and the increment interval of the introduced time error [13]. If the cumulated offset error of all slaves as reported by their SCSM exceeds the previously determined threshold in at least one synchronization cycle, an alarm is raised regardless of the TSN type used (see Algorithm 2).
**Algorithm 2:** Time source attack detection1**Input**: activeBucket2**Output**: Attack detected (Y/N)3*offset*:Hash table containing slaves’ offset values extracted from SCSM 4*offsetMax*:Hash table containing the maximum offset value of each slave (offset_max_)5*offsetMin*:Hash table containing the minimum offset value of each slave (Offset_min_)6*counter*:counter counts how many slaves exceed its Offset_max_ and Offset_min_7*GrandID*:Hash table containing slaves’ *grandID* extracted from SCSM8*GrandID2*:the *grandID* as stored in the database9*IP*:A list containing the *slaveID* extracted from SCSM 10*counter* = 011**for** each *i in IP* **do**12      **if** *offset[i]* > *offsetMax[i]*
**or**
*offset[i] < offsetMin[i]      ***then**13           *counter* = *counter* + 114      **end if**15      **if**  *GrandID[i]* < > *GrandID2***   then**16           c*ounter* = *IP.size +* 117      **end if**18**end for**19**if** *counter* = *IP.size*
**then**20    **print** “Time Source attack or BMCA Attack”21**else if***counter* = *IP.size +* 1 **then**22**    print** “BMCA Attack”23**end if**24

2. BMCA attack

In addition to the above method, a BMCA attack may also coincide with a changing *grandID* (the ID of the new GM), which is checked for by the TSN. If an attacker is able to spoof the original GM’s *grandID*, an attack will be incorrectly labelled as a time source degradation attack.

However, an attacker can exploit the vulnerability of TSN Type A by manipulating the GM timestamp as well as keeping offset errors within the previously established offset boundaries. Such an attack can be implemented by introducing a very small increment to *t1* over a long attack interval so that the calculated offset remains in the normal range. In addition, the BMCA attack detection will fail if the rogue grandmaster uses the same identity and network location as the old grandmaster, and both of them have the same accuracy and frequency at the beginning of the attack (i.e., the rogue master is synchronized to the old grandmaster). Nevertheless, the Type-B TSN is able to detect such attacks by comparing its compromised synchronized PTP clock against its local clock. It raises an alarm if the difference between both exceeds T_Δ_.

3. Comparison with the State-of-the-Art

Table 1 shows a comparison of existing attack detection methods in terms of detectable attacks and their limitations. Annex P prong D can detect packet delay manipulation attacks, DoS attacks, BMCA attacks, and packet content manipulation attacks, but is not able to detect the other PTP attacks, including replay attacks, spoofing attacks, and time source degradation attacks. In addition, it does not provide a mechanism to identify the attack-type, or state how an alarm can be triggered. Moussa [21] provided only a mechanism to detect the delay attack while the other attacks are ignored. In addition, the provided mechanism does not cover all delay attack scenarios. For example, it works efficiently when an attacker delays *Sync* messages but fails when the attacker delays *Delay_Req* messages. Moussa [22] provided a mechanism to detect attacks depending on attacker locations, i.e., GM, TC, slave, and a communication path (e.g., delay attack). However, the provided mechanism relies on a redundant time source that is vulnerable itself to an attack (e.g., an attacker simultaneously manipulates all time references in a network). Moreover, an attacker can manipulate timestamps sent by slaves to the redundant GM to hide the attack. Finally, Ullmann [24] provided only a mechanism to detect the delay attack while the other attacks are ignored. Furthermore, the suggested solution is prone to raise false alarms.

In comparison to these methods, the proposed method is able to detect all potential PTP attacks as outlined in [7], as well as the likely location of the attacker within the network. In addition, the TSN itself as well as all communication with slaves have been hardened against attacks that compromise the operation of the TSN. In other words, the TSN has overcome all the limitations of the other approaches.

## 4. The Testbed and Experiments

### 4.1. Testbed

**Figure 5:**
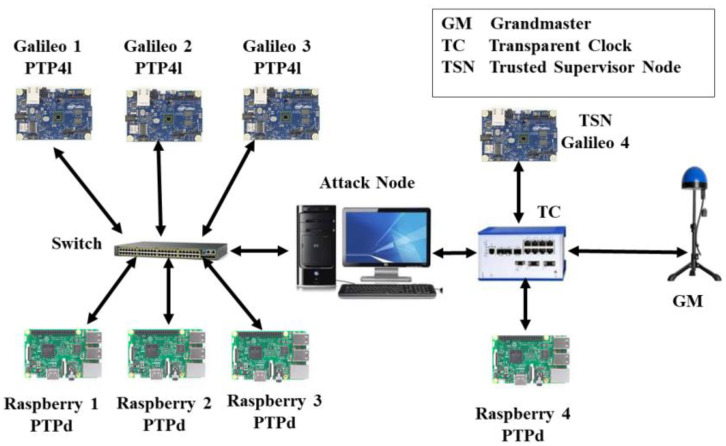
The experimental testbed.

The experimental testbed to validate the TSN concept is shown in Figure 5. It consists of the following hardware and software.

#### 4.1.1. Grandmaster Clock

The testbed has only one time source that acts as a grandmaster clock, an Omicron OTMC 100-antenna-integrated PTP Grandmaster Clock.

#### 4.1.2. Slave Clocks

There are seven slave clocks in the testbed comprising three Intel Galileo Gen 1 and four Raspberry Pi 3 model B. The PTP4l daemon is used by Galileo devices, whereas PTPd is used by Raspberry devices. A separate Galileo 4 implements a Type-A TSN. Both slaves run a Linux OS.

#### 4.1.3. Intermediate Nodes

The testbed consists of both PTP-aware and PTP-unaware intermediate nodes. The latter comprises a Cisco 16-port switch, whereas the former is made of a Hirschmann RSP20 transparent clock.

#### 4.1.4. Attack Node

Since manipulating the switches’ or TC’s firmware to implement an attack is a tedious and complex task, the more flexible option of a programmable MitM attacker has been used. This device is a Linux computer with two network interfaces, which are connected by a bridge using the Linux ebtables tool. A user-space program selectively manipulates incoming PTP messages from the input port, before forwarding them to the output port. While the device is placed between the GM and the targeted slaves, it effectively behaves like an attacker located on the network infrastructure hardware, with the exact location being picked by the LCA algorithm [13].

#### 4.1.5. Software

Both the slaves’ monitor program and data concentrator program on the TSN are written in C. The slave monitor program works with both aforementioned PTP slave daemons and generates SCSM packets utilizing the payload from grandmaster synchronization messages (i.e., *syncID*, *t1*, *c2*, *grandID*, and *syncInterval*), a slaves’ IP address, offset and delay values provided by the daemon, and calculates the estimated timestamp *EMT*.

All hardware in the testbed is connected by CAT5e Ethernet cables with a data rate of 1000 Mbps. Since the slaves do not support the P2P delay mechanism and hardware timestamping, the E2E delay mechanism with two-step mode operation are used. In addition, the log sync interval is set to 0 in all conducted experiments, which makes the grandmaster to send *Sync*/*Follow_Up* messages every second. In addition, all conducted experiments were conducted using IEEE 1588–2008. It is worth noting that these configurations are used by many profiles, such as the IEEE 1588 default profile [1], SMTPE ST-2059–2 profile [33], and ITU-T Telecom G.8275.2 profile [34].

### 4.2. Experiments

Several experiments were conducted to examine the TSN’s ability to detect various PTP attack types using the setup shown in Figure 5.

#### 4.2.1. Baseline Determination Stage

The TSN requires predefined settings to build and determine the slaves’ baselines parameters. Table 2 shows the TSN settings that were used for the experiments. A Z-score boundary of 3.6 standard deviations in conjunction with a buffer size of 100 values provides a 1% significance level for acceptable offset values [31]. N_SCSM_ is set to 10.

The testbed was powered up for 15 min before the baseline determination stage. As shown in Table 3, calculated Delay_max_ and Delay_min_ values vary depending on the PTP daemon type used, as each daemon type applies a different delay filter [13], and the slave location within the network (i.e., the presence of a normal switch in the sync packet path causes a larger offset range). C_max_ is determined by the peak traffic volume that passes the TC.

#### 4.2.2. Synchronization Attack Detection

In the conducted experiments, the TSN is exposed to all potential PTP attacks as listed in [7], i.e., packet content manipulation, packet removal, packet delay manipulation, spoofing, replay, and Denial of Service (DoS) attacks. All experiments begin with a 5-min phase of normal time synchronization operation.

1. Packet content manipulation attack
  (a) The attack node (see Figure 5) intercepts all *Sync* messages and increments *t1* by 10 µs. The TSN immediately detects the attack and raises an alarm, as the unaffected Raspberry 4 reports a different *t1*. Since the TSN picked Galileo 1 as the baseline of *t1* comparison, the LCA algorithm incorrectly returns the TC as a probable attack location as already explained above.
  (b) The experiment is repeated by adding an incremental 20 µs to the *correctionField* value (*c2*) with every synchronization cycle (i.e., every second). The value of *c2* increases from 3.4 ms to 12.7 ms 465 s later, at which point the TSN detected the attack. Here, the *c2* value of Galileo 1, Galileo 2, Galileo 3, Raspberry 1, Raspberry 2, and Raspberry 3 (see Table 3) are manipulated and subsequently exceed their C_max_ (see Table 4), while *c2* of Raspberry 4 remains within normal range. The LCA algorithm returns the switch as the source of the attack.
2. Replay and spoofing attack
  (a) The attack node copies each *Sync*/*Follow_Up* message and replays them after 1 ms. The TSN immediately identifies the attack as a replay/spoofing attack and determines the attacker’s probable location (i.e., the switch), as the TSN received duplicated SCSMs from all slave clocks except for Raspberry 4.
  (b) In a second experiment, each *Sync*/*Follow_Up* message is copied and the *preciseOriginTimestamp* content of *Follow_Up* messages is increased by 5 ms, before the copied messages are sent to their destination, therefore simulating the spoofing attack. Again, the TSN recognizes and classifies the attack correctly, while the LCA algorithm returns the correct attacker location.
3. Packet delay manipulation attack

The attack node intercepts each *Sync* message and holds it for 50 µs. The TSN detects the attack after all buffered delay values (i.e., circular buffer of size N_B_) are affected by the increment (i.e., 100 s later) and after N_SCSM_ is exceeded. The new delay averages of Galileo 1, Galileo 2, Galileo 3, Raspberry 1, Raspberry 2, and Raspberry 3 are 538.7 µs, 541.1 µs, 543.1 µs, 276.8 µs, 343.4 µs, and 295.2 µs, respectively, at the attack detection point (Table 4). The LCA correctly identifies the switch as the potential attacker location.

4. Denial of service (DoS) attack

The attack node intercepts and removes all *Follow_Up* messages. As a result, the manipulated slaves (excluding Raspberry 4) stop sending their data to the TSN device, which is picked up by the TSN after N_SCSM_ missed synchronization cycles. The LCA algorithm returns correctly the switch as the potential attacker location.

5. Unknown attack

It is possible that an attacker may develop a PTP attack strategy that fools some parameters used in the detection process (e.g., N_SCSM_) that leads to hide the attack type. However, all previous attacks are also detected by the *EMT* comparison when Galileo 1, Galileo 2, Galileo 3, Raspberry 1, Raspberry 2, and Raspberry 3 clocks are behind/ahead of the GM/Raspberry 4 clock by T_Δ_. To highlight the importance of the *EMT* comparison, the *correctionField* manipulation experiment (i.e., packet content manipulation attack—experiment no. 2) is repeated with pausing the attack before the affected SCSMs exceed N_SCSM_. In this case, the attacker deceives the *correctionField* comparison, but the *EMT* comparison triggers an alarm when the difference between the Raspberry 4 *EMT* and the other slaves’ *EMTs* exceeds T_Δ_ (i.e., 1 ms). Since the TSN picked Galileo 1 as the baseline of the *EMT* comparison, the LCA algorithm incorrectly returns the TC as a probable attack location.

6. Simultaneous attacks

The experiments of manipulating *correctionField* value (*c2*) and manipulating the delay average are simultaneously repeated. As a result, the TSN detects that Galileo 1, Galileo 2, Galileo 3, Raspberry 1, Raspberry 2, and Raspberry 3 were manipulated by the packet content manipulation attack and the packet delay manipulation attack via the switch.

#### 4.2.3. Time Source Attack Detection

Time source degradation attack

(a) The attacking node is moved between the GM and the TC in order to intercept and manipulate the *t1* value in all *Follow_Up* messages, which affects all slave clocks, therefore simulating the time source degradation attack. The attacking node increments the *preciseOriginTimestamp* value by 200 µs every 5 min. The TSN immediately detects the attack, as all slaves’ offsets exceeded their baselines values (see Table 4).
(b) The same experiment is repeated with an increment of 10 µs every 15 min. In contrast, the TSN is unable to detect this attack as all slave offsets remain with their normal range.
(c) Galileo 1 is configured to be the new grandmaster by changing the *priority1* value to be smaller than the original GM *priority1* value. As a result, all slave clocks show abnormal offset values (see Table 4), and subsequently, the TSN detects the attack.

## 5. Summary of Results and Discussion

Table 4 provides a summary of all conducted attacks. The data shown represent parameter values sent by slaves during a specific synchronization cycle, when the TSN detects the attack. All slaves that are located between the switch and the attacking node are manipulated by the packet content manipulation attack, replay/spoofing attack, packet delay manipulation attack, DoS attack, or unknown attack; therefore, they exceed at least one of their baseline values, while the parameters of Raspberry 4, which is out of synchronization packet attacks scope, remain within the normal range. In contrast, all slaves’ offsets (including Raspberry 4) are affected by the time source attack 1 and 3. Although the time source attack 2 does also manipulate all slaves, their offsets remain between Offset_max_ and Offset_min_, since the attacker introduced a small increment for the *t1* value (i.e., 10 µs) over a long attack interval (i.e., 15 min); therefore, it did not create instantaneous spike of offset errors in all slaves. To tackle this problem, the Type-B TSN is introduced. The Type-B TSN calculates the difference between its TSN clocks, i.e., the PTP clock and the local clock, and raises an alarm if the difference exceeded T_Δ_. As a result, the Type-B TSN is able to detect any attack. It can be noted that keeping the PTP clock of a Type-B TSN synced to the GM does not limit the TSN role, as the TSN PTP clock will give an opportunity to detect the most silent attack, i.e., time source attack, as described above.

The TSN may fail to identify intermittently paused attacks, which never exceed the N_SCSM_ threshold, as done in the unknown attack experiment. In addition, the LCA may incorrectly return the probable location of the attacker if a manipulated parameter is chosen as baseline value, as happened in the packet content manipulation attack (experiment no.1) or the unknown attack experiment.

In addition, a high fluctuation of delay values during the baseline determination stage leads to a high delay range, giving an attacker more flexibility to manipulate PTP packets without being detected (i.e., the better managed a network is, the tighter the margins for delay and offset values are, the smaller the N_SCSM_ threshold can be chosen, making it harder for an attacker to manipulate PTP packets).

However, all of these limitations do not limit the TSN’s ability (the Type-B in particular) in detecting PTP attacks, as the Type-B TSN will use its own time reference as a benchmark for detecting attacks.

## 6. Conclusions and Future Work

This paper introduces two algorithms to detect and determine the type and location of potential PTP attacks using two different types of trusted supervisor nodes (TSN). It follows the ideas of prong D as specified in IEEE 1588–2019 Annex P. Both algorithms collect baseline data over a training period, thereby determining slave-specific boundaries of clock parameters. As such, the proposed methods provide another layer of defense against PTP attacks.

The first algorithm detects attacks that manipulate PTP synchronization packets in transit, thereby manipulating downstream slave clocks, which subsequently show different characteristics compared to unaffected nodes. The second algorithm tests for attacks that originate at the time synchronization source. Both algorithms use baseline data from each slave clock which are collected during an initial training phase.

The experiments show the ability of the TSN to detect the packet content manipulation attack, replay and spoofing attack, packet delay manipulation, and DoS attack, including the packet removal attack as an example of a DoS attack.

While the Type-A TSN is able to detect all PTP attacks that affect the parameters sent through SCSM, the Type-B TSN can detect abnormalities even if the sent parameters remain within their normal range, allowing to detect the most silent attack, i.e., the time source attack.

The TSN uses the lowest common ancestor method (LCA) to determine the probable location of the attacker. However, the execution of LCA may return an incorrect result with packet content manipulation attack (*t1* attack) and the unknown attack.

Instable delay paths between the GM and its slaves result in high fluctuations in delay values and therefore increase the delay and offset range of a slave. Such behavior complicates the detecting a delay attack or makes all time source attacks undetectable by the Type-A TSN. Therefore, the smaller the delay/offset range is, the earlier an attack is detected.

While the TSN provides only detection methods, further lines of defense must be considered to prevent internal PTP attacks, for example, by hardening network infrastructure components via TPM.

## Figures and Tables

**Table 1:** Efficiencies and limitations of existing attack detection methods.

Proposed Detection Method	Detectable Attacks	Limitations
Annex P (Prong D)	Delay attack DoS attack BMCA attack Packet content manipulation attack	Does not provide a mechanism to identify an attack type nor how an alarm can be triggered. Replay, spoofing, time source degradation attacks are ignored.
Moussa [21]	Delay attack	Other attacks are not addressed. Does not provide a detection method against delaying a PTP packet from slave to master path.
Moussa [22]	Attacks on GM Attacks on TC Attacks on slave Delay attack	Relies on a redundant time source that is vulnerable itself to an attack. Requires sending timestamps from slaves to the redundant time source which may be prone to delay attack or packet content manipulation attack.
Ullmann [24]	Delay attack	Does not cover other attacks. Low specificity.

**Table 2:** TSN settings.

Fields	Values
Database Building Time C_P_	24 h
circular buffer size N_B_	100 entries
Z-score-boundary to detect suspicious delay values Z_max_	3.6 standard deviations
The maximum acceptable timestamp difference T_Δ_	1 millisecond
SCSM Attack Threshold N_SCSM_	10 packets

**Table 3:** Baseline parameters under normal conditions.

Devices	Delay_max_	Delay_min_	Offset_max_	Offset_min_	C_max_
Galileo 1	538.2 µs	495.1 µs	122.6 µs	−141.8 µs	11.6 ms
Galileo 2	540.7 µs	496.4 µs	144.2 µs	−102.2 µs	11.6 ms
Galileo 3	542.9 µs	495.4 µs	128.9 µs	−140.5 µs	11.6 ms
Raspberry 1	276.7 µs	256.4 µs	111.6 µs	−109.5 µs	11.6 ms
Raspberry 2	343.3 µs	239.4 µs	83.2 µs	−68.6 µs	11.6 ms
Raspberry 3	295.1 µs	241.8 µs	62.4 µs	−82.4 µs	11.6 ms
Raspberry 4	89.4 µs	82.6 µs	27.2 µs	−23.3 µs	11.6 ms

**Table 4:** Experimental Results Summary.

Devices	Packet Content Manipulation Attack Experiment 1	Packet Content Manipulation Attack Experiment 2	Replay and Spoofing Attack 1	Replay and Spoofing Attack 2	Packet Delay Manipulation Attack	DoS Attack	Unknown Attack	Time Source Attack 1	Time Source Attack2	Time Source Attack 3
	Parameter Used to Detect the Attack									
	*t1*	*c2*	slaveID	slaveID	Delay Average	SCSM	EMT	Offset	Offset	Offset
Galileo 1	1608980367. 866912616	12.7 ms	Duplicated	Duplicated	538.7 µ	No SCSMs Received	1609081350. 872350145	215 µs	−43 µs	GM
Galileo 2	1608980367. 866912616	12.7 ms	Duplicated	Duplicated	541.1 µ	No SCSMs Received	1609081350. 872024267	190 µs	−34 µs	231 µs
Galileo 3	1608980367. 866912616	12.7 ms	Duplicated	Duplicated	543.1 µ	No SCSMs Received	1609081350. 872141431	257 µs	−13 µs	221 µs
Raspberry 1	1608980367. 866912616	12.7 ms	Duplicated	Duplicated	276.8 µ	No SCSMs Received	1609081350. 872816678	206 µs	5 µs	258 µs
Raspberry 2	1608980367. 866912616	12.7 ms	Duplicated	Duplicated	343.4 µ	No SCSMs Received	1609081350. 872546247	214 µs	10 µs	250 µs
Raspberry 3	1608980367. 866912616	12.7 ms	Duplicated	Duplicated	295.2 µ	No SCSMs Received	1609081350. 872394132	215 µs	7 µs	251 µs
Raspberry 4	1608980367. 867112616	9.2 ms	-	-	84.7 µ	SCSMs Received	1609081350. 873466305	183 µs	22 µs	290 µs

## Data Availability

The data presented in this study are available from the corresponding author upon request.

## References

[1] (2008). IEEE Standard for a Precision Clock Synchronization Protocol for Networked Measurement and Control Systems.

[2] Alghamdi W., Schukat M. Advanced methodologies to deter internal attacks in PTP time synchronization networks. Proceedings of the 2017 28th Irish Signals and Systems Conference (ISSC).

[3] Itkin E., Wool A. (2020). A security analysis and revised security extension for the precision time protocol. IEEE Trans. Dependable Secur. Comput..

[4] (2020). IEEE Standard for a Precision Clock Synchronization Protocol for Networked Measurement and Control Systems.

[5] Shereen E., Bitard F., Dán G., Sel T., Fries S. Next Steps in Security for Time Synchronization: Experiences from implementing IEEE 1588 v2.1. Proceedings of the 2019 IEEE International Symposium on Precision Clock Synchronization for Measurement, Control, and Communication (ISPCS).

[6] Mizrahi T. Time synchronization security using IPsec and MACsec. Proceedings of the International IEEE Symposium on Precision Clock Synchronization for Measurement Control and Communication.

[7] Alghamdi W., Schukat M. (2021). Precision time protocol attack strategies and their resistance to existing security extensions. Cybersecurity.

[8] Mizrahi T. (2014). Security Requirements of Time Protocols in Packet Switched Networks. https://tools.ietf.org/html/rfc7384.

[9] Estrela P.V., Neusüß S., Owczarek W. Using a multi-source NTP watchdog to increase the robustness of PTPv2 in financial industry networks. Proceedings of the Precision Clock Synchronization for Measurement, Control, and Communication (ISPCS), 2014 IEEE International Symposium on.

[10] Shpiner A., Revah Y., Mizrahi T. Multi-path Time Protocols. Proceedings of the 2013 IEEE International Symposium on Precision Clock Synchronization for Measurement, Control and Communication (ISPCS) Proceedings.

[11] Koskiahde T., Kujala J. PTP monitoring in redundant network. Proceedings of the 2016 IEEE International Symposium on Precision Clock Synchronization for Measurement, Control, and Communication (ISPCS).

[12] Dalmas M., Rachadel H., Silvano G., Dutra C. Improving PTP robustness to the byzantine failure. Proceedings of the 2015 IEEE International Symposium on Precision Clock Synchronization for Measurement, Control, and Communication (ISPCS).

[13] Alghamdi W., Schukat M. (2020). Cyber Attacks on Precision Time Protocol Networks—A Case Study. Electronics.

[14] Garner G.M. (2008). IEEE 1588 Version 2. ISPCS Ann. Arbor.

[15] Önal C., Kirrmann H. Security improvements for IEEE 1588 Annex K: Implementation and comparison of authentication codes. Proceedings of the Precision Clock Synchronization for Measurement Control and Communication (ISPCS), 2012 International IEEE Symposium on.

[16] Pathan Y., Dalvi A., Pillai A., Patil D., Reed D. (2014). Analysis of selective packet delay attack on IEEE 1588 Precision Time Protocol. u. Colo. ITP Proj..

[17] Maftei D., Bartos R., Noseworthy B., Carlin T. Implementing proposed IEEE 1588 integrated security mechanism. Proceedings of the 2018 IEEE International Symposium on Precision Clock Synchronization for Measurement, Control, and Communication (ISPCS).

[18] Han M., Crossley P. Vulnerability of IEEE 1588 under Time Synchronization Attacks. Proceedings of the 2019 IEEE Power & Energy Society General Meeting (PESGM).

[19] Neyer J., Gassner L., Marinescu C. Redundant Schemes or How to Counter the Delay Attack on Time Synchronization Protocols. Proceedings of the 2019 IEEE International Symposium on Precision Clock Synchronization for Measurement, Control, and Communication (ISPCS).

[20] Dadheech K., Choudhary A., Bhatia G. De-Militarized Zone: A Next Level to Network Security. Proceedings of the 2018 Second International Conference on Inventive Communication and Computational Technologies (ICICCT).

[21] Moussa B., Debbabi M., Assi C. (2018). A Detection and Mitigation Model for PTP Delay Attack in an IEC 61850 Substation. IEEE Trans. Smart Grid.

[22] Moussa B., Kassouf M., Hadjidj R., Debbabi M., Assi C. (2020). An Extension to the Precision Time Protocol (PTP) to Enable the Detection of Cyber Attacks. IEEE Trans. Ind. Inform..

[23] Baize E. (2012). Developing Secure Products in the Age of Advanced Persistent Threats. IEEE Secur. Priv..

[24] Ullmann M., Vögeler M. Delay attacks—Implication on NTP and PTP time synchronization. Proceedings of the 2009 International Symposium on Precision Clock Synchronization for Measurement, Control and Communication.

[25] Moradi M., Jahangir A.H. (2021). A new delay attack detection algorithm for PTP network in power substation. Int. J. Electr. Power Energy Syst..

[26] Lisova E., Gutiérrez M., Steiner W., Uhlemann E., Åkerberg J., Dobrin R., Björkman M. (2016). Protecting Clock Synchronization: Adversary Detection through Network Monitoring. J. Electr. Comput. Eng..

[B27-sensors-22-03671] 27.Cyber-Security of Time-Aware Cyber-Physical Systems. in Re-Industrialisation of the EU, 2016.

[28] Limited E.T. (2016). IEEE 1588 PTP Clock Synchronization over a WAN Backbone. https://www.endace.com/ptp-timing-whitepaper.pdf.

[29] Shannon J., Melvin H., Ruzzelli A.G. Dynamic flooding time synchronisation protocol for WSNs. Proceedings of the 2012 IEEE Global Communications Conference (GLOBECOM).

[30] Schukat M., Castilla P.C., Melvin H., Hu F. (2016). Trust and Trust Models for the IoT. Security and Privacy in Internet of Things (IoTs): Models, Algorithms, and Implementations.

[31] Grubbs F.E., Beck G. (1972). Extension of sample sizes and percentage points for significance tests of outlying observations. Technometrics.

[32] Bender M.A., Farach-Colton M., Pemmasani G., Skiena S., Sumazin P. (2005). Lowest common ancestors in trees and directed acyclic graphs. J. Algorithms.

[33] (2015). ST 2059-2:2015-SMPTE Standard-SMPTE Profile for Use of IEEE-1588 Precision Time Protocol in Professional Broadcast Applications.

[34] (2020). T.U. G.8275.2: Precision Time Protocol Telecom Profile for Time/Phase Synchronization with Partial Timing Support from the Network. https://www.itu.int/rec/T-REC-G.8275.2-202003-I/en.

